# Editorial: Complement and Immunotherapeutics

**DOI:** 10.3389/fimmu.2021.663459

**Published:** 2021-02-24

**Authors:** Marcin Okrój, Elena Volokhina

**Affiliations:** ^1^Intercollegiate Faculty of Biotechnology, University of Gdańsk and Medical University of Gdańsk, Gdańsk, Poland; ^2^Department of Pediatric Nephrology, Amalia Children's Hospital, Radboud Institute for Molecular Life Sciences, Nijmegen, Netherlands; ^3^Department of Laboratory Medicine, Radboud University Medical Center, Nijmegen, Netherlands

**Keywords:** complement, immunotherapy, rituximab, eculizumab, factor H, antibodies

The complement system was discovered more than 100 years ago as an ancient defense system that prevents pathogens' invasion. For a long time, the complement was considered a scientifically interesting system with limited physiological relevance. However, it regained interest in the last decades when thousands of publications on its new role in health and disease appeared. The loss of proper control on complement activity became an acknowledged etiological factor in numerous autoimmune/inflammatory diseases. The discovery of complement receptors revealed that this system is not only an implement to kill microbes but an essential modulator of the immune responses, metabolism, angiogenesis, tissue repair and development, and many other processes influencing the overall body homeostasis (1–3). Additional interest in complement as an effector mechanism of therapeutic antibodies appeared when rituximab, the first antitumor antibody, was introduced into the clinics in 1997 (4) and a decade later when the first complement-specific drug, eculizumab was approved (5). Engagement of more than 40 proteins either as structural elements of the complement cascade or its regulators as well as crosstalk with other pathways creates a sophisticated network of interactions that keep a delicate balance between the deleterious and protective nature of the complement. Therefore, it is not surprising that on one hand markers of complement activation emerge as diagnostic and prognostic tools (6), and on the other hand increasing number of complement targets for potential therapeutic intervention appears, which are currently being exploited by registered drugs or compounds that are under evaluation in clinical trials (7).

This Research Topic entitled “Complement and Immunotherapeutics” presents a collection of articles describing a broad range of developments in the field of complement-targeting drugs.

The article by Urwyler et al. presents the results of an uncontrolled series of cases, where patients suffering from SARS-CoV-2 infection were treated with conestat alpha—the recombinant C1 inhibitor. Out of five patients with severe COVID-19 pneumonia enrolled into the therapeutic scheme, four patients achieved immediate recovery, and one needed mechanical ventilation, however, all patients finally recovered. Conestat showed good tolerability. Despite certain limitations of this study, the results encourage to perform of a controlled clinical trial.

The article by Zelek and Morgan introduces a novel monoclonal antibody targeting C7 and C5b-7 complex. Such antibody was successfully tested in both *in vitro* systems and *in vivo* models of C7-deficient mouse reconstituted with human C7 or rat model. Such an innovative therapeutic approach allows targeting of a membrane attack complex formation, a very late step of the cascade, leaving all upstream steps intact to protect the host from pathogens and clean up cell debris.

A novel therapeutically useful concept was presented by Banda et al. and involves natural IgM antibodies (NAbs). These antibodies are known to recognize injury-associated epitopes and may contribute to autoimmunity. However, when engineered, they may be employed to deliver complement regulators to the site of injury. In this study fusion protein consisting of NAb-derived fragment and mouse complement inhibitor Crry was shown to decrease clinical disease activity in a mouse model of arthritis. Another fusion construct of factor H (FH) CCP domains 18–20 with Fc fragment of human IgG1 applicable in combating *Neisseria gonorrhoeae* infection was described by. *Shaughnessy et al*. Point mutation introduced in CCP19 disabled lysis of human erythrocytes but left bactericidal potential. Production of recombinant protein in a high-yield plant system was described and the efficacy of the product was proven in a mouse model. Staying in the field of complement inhibitors, de Boer et al. have reviewed the role of complement regulation in oncological, anemic, renal, eye, and neurologic pathologies. The authors also discussed the potential of either full-length or engineered inhibitors and antibodies targeting complement regulatory proteins in the context of the abovementioned diseases and unmet clinical needs. Not only disruption of complement regulation but also deficiency of essential complement components may lead to autoimmunity. An example is C1q deficiency, which plays an important role in the development of systemic lupus erythematosus (SLE). The comprehensive review by *Hosszu et al*. discusses the impact of the C1q/C1qR axis in monocyte-to-dendritic cell differentiation and how the distortion of this axis leads to disease. Moreover, the authors deliberate on possible therapeutic strategies to attenuate pro-inflammatory response in SLE patients.

Two articles in the collection are dedicated to anti-CD20 antibodies. Bondza et al. dissected the mechanistic details of the interaction between clinically approved anti-CD20 mAbs (rituximab, ofatumumab, and obinutuzumab) and their target. The authors analyzed the kinetics of interactions and discussed them in the context of complement activation and complement-dependent cytotoxicity. Felberg et al. analyzed the cytotoxic potential of sera as well as the levels of complement activation markers and retention of rituximab in patients treated for lymphoproliferative disorders. The publication concludes that monitoring of the complement system status and measurement of cell-free rituximab during therapy may be valuable for clinicians and become the step forward to personalized treatment for the patient. The additional potentially relevant parameter in the course of immunotherapy is chronic, low-level complement activation. Naseraldeen et al. described complement-activating IgG hexamers in sera from chronic lymphocytic leukemia (CLL) patients and the pivotal role of alpha-2 microglobulin in their formation.

A lot of attention is given to the alternative complement pathway (AP) in the context of kidney diseases, as uncontrolled propagation of AP often leads to severe damage and eventually loss of kidney function. Eculizumab was the first complement medication approved for the treatment of atypical hemolytic uremic syndrome (aHUS). The two case-reports illustrate the heterogeneity of this disease and challenge for clinicians to establish a correct diagnosis and treatment scheme and the necessity of monitoring disease progression and complement status. Lumbreras et al. describe a patient with a very mild manifestation of aHUS. The aHUS diagnosis was established when low C3 levels and C3 genetic variant was detected. The mild phenotype may be explained by the absence of MCP risk polymorphisms in this patient. While in good health during publication, the patient should be closely monitored for signs of aHUS relapse to initiate complement therapy on time. Bouwmeester et al. described a case of a severely affected patient with aHUS that under eculizumab treatment has shown high intra-patient variability in the serum drug levels and CH50, and decline of renal function. This case underscores the need for continuous monitoring of patients under treatment for drug levels and complement status.

*Lammerts et al*. studied the nature of properdin binding to proximal tubular endothelial cells and found that this process and subsequent AP activation can be blocked by the recombinant tick protein Salp20. Intravascular hemolysis and release of heme may lead to acute kidney injury. *Merle et al*. assessed the role of the main fluid-phase inhibitor of AP, FH, in kidney protection from hemolysis-mediated damage. Authors postulate that FH-based approaches can be exploited as promising therapeutic strategies. The work by *Koopman et al*. focuses on C5b-9 deposition in the kidney. Nowadays, therapies of renal diseases include blockers of the terminal complement pathway (e.g., eculizumab), but it is not entirely clear how C5b-9 contributes to pathological processes since such staining is not routinely performed. Comparison between healthy and diseased kidneys, correlation with other histological lesions and clinical data, and potential prognostic value of C5b-9 deposits are discussed.

Chronic inflammation is observed in women suffering from endometriosis. Such inflammatory status may stem from pathological complement activation fueled by aberrant regulation of the cascade. *Agostinis et al*. discussed the prospect of complement inhibition as a novel therapeutic approach in endometriosis.

La-Beck et al. reviewed the link between adverse effects of nanoparticle-based drugs and activation of the complement system. Complement activation on nanoparticles generates either opsonins and their cleavage products or release of anaphylatoxins. Both processes can diminish the efficacy of nano-encapsulated anticancer therapeutics by uptake of nanoparticles by immune cells and creation of pro-tumor microenvironment via C5aR-dependent signaling. Understanding a complex network of nanoparticles, complement, and drug pharmacology will aid the improvement of cancer nanomedicines.

In summary, the current Research Topic is a collection of 16 original and review articles, which describe novel candidates for complement-related drugs, mechanistic insights of drug-target interaction, target cell sensitivity, nanoparticles-based complement medications, diagnostics, complement genetic screening, as well as complement involvement in the course and treatment of diseases such as systemic lupus erythematosus (SLE), endometriosis, and a wide spectrum of kidney diseases. The articles highlight novelty and future perspectives of recent developments in these fields.

## Author Contributions

MO and EV wrote the manuscript and agreed on its final version. Both authors contributed to the article and approved the submitted version.

## Conflict of Interest

The authors declare that the research was conducted in the absence of any commercial or financial relationships that could be construed as a potential conflict of interest.
